# Novel irreversible electroporation ablation (Nano-knife) versus radiofrequency ablation for the treatment of solid liver tumors: a comparative, randomized, multicenter clinical study

**DOI:** 10.3389/fonc.2022.945123

**Published:** 2022-09-29

**Authors:** Xiaobo Zhang, Xiao Zhang, Xiaoyi Ding, Zhongmin Wang, Yong Fan, Guang Chen, Xiaokun Hu, Jiasheng Zheng, Zhixiao Xue, Xiaofeng He, Xin Zhang, Yingtian Wei, Zhongliang Zhang, Jing Li, Jie Li, Jie Yang, Xiaodong Xue, Li Ma, Yueyong Xiao

**Affiliations:** ^1^ Department of Radiology, First Medical Center, Chinese People's Liberation Army General Hospital, Beijing, China; ^2^ Chinese PLA Medical School, Beijing, China; ^3^ Department of Interventional Radiology, Ruijin Hospital, Shanghai Jiao Tong University School of Medicine, Shanghai, China; ^4^ Department of Medical Imaging, Tianjin Medical University General Hospital, Tianjin, China; ^5^ Department of Radiology, Tianjin First Central Hospital, Tianjin, China; ^6^ Department of Interventional Radiology, Affiliated Hospital of Qingdao University, Qingdao, China; ^7^ Center of Interventional Oncology and Liver Diseases, Beijing Youan Hospital, Beijing, China; ^8^ School of Biomedical Engineering and Technology, Tianjin Medical University, Tianjin, China; ^9^ Department of Radiology, Characteristic Medical Center of Chinese People’s Armed Police Force, Tianjin, China; ^10^ Department of Anesthesiology, First Medical Center, Chinese People’s Liberation Army General Hospital, Beijing, China

**Keywords:** irreversible electroporation-based ablation (IRE), ablation, radiofrequency, liver cancer, hepatocellular carcinoma

## Abstract

Irreversible electroporation (IRE) is a soft tissue ablation technique that uses short electrical fields which induce the death of target cells. To evaluate the safety and efficacy of an IRE-based device compared to regular radiofrequency ablation (RFA) of solid liver tumors, in this multicenter, randomized, parallel-arm, non-inferiority study, 152 patients with malignant liver tumors were randomized into IRE (n = 78) and RFA (n = 74) groups. The primary endpoint was the success rate of tumor ablation; the secondary endpoints included the tumor ablation time, complications, tumor recurrence rates and treatment-related adverse events (TRAE). The success rate of tumor ablation using IRE was 94.9% and was non-inferior to the RFA group (96.0%) (P = 0.761). For the secondary endpoints, the average ablation time was 34.29 ± 30.38 min for the IRE group, which was significantly longer than for the RFA group (19.91 ± 16.08 min) (P < 0.001). The incidences of postoperative complications after 1 week (P = 1.000), 1 month (P = 0.610) and 3 months (P = 0.490) were not significantly different between the 2 groups. The recurrence rates of liver tumor at 1, 3 and 6 months after ablation were 0 (0.0%), 10 (13.9%) and 10 (13.3%) in the IRE group and 2.9%, 7.3% and 19.7% in the RFA control group (all P > 0.05), respectively. For safety assessments, 51 patients experienced 191 AEs (65.4%) in the IRE group, which was not different from the RFA group (73.0%, 54/184) (P = 0.646). In 7 IRE patients, 8 TRAEs (7.9%) occurred, the most common being edema of the limbs (mild grade) and fever (severe grade), while no TRAEs occurred in the RFA group. This study proved that the excellent safety and efficacy of IRE was non-inferior to the regular radiofrequency device in ablation performance for the treatment of solid liver tumors. **Clinical trial registration**: Chinese Clinical Trial Registry: ChiCTR1800017516

## 1 Introduction

Tumor ablation techniques, including the use of radiofrequency, microwaves and cryoablation, have been widely adopted in clinical practice (1–6). Radiofrequency ablation (RFA) requires the placement of an ablation probe into the target tissue with the formation of a certain range of ellipsoidal ablation zone around the lesion. On the other hand, irreversible electroporation (IRE) positions the probe on the outer edge of the interface between the tumor and normal tissue, and uses high-voltage electrical pulses between two probe pairs to achieve ablation. Many preclinical studies of IRE have provided evidence that vessels, bile ducts and adjacent tissues in the ablation zone can be preserved. Due to its advantages of small treatment trauma, rapid recovery, avoidance of the “heat sink effect” due to thermal ablation, treatment of tumors in the vicinity of vital structures and short hospital stays, the introduction of IRE has been well received by many patients and physicians. After the US Food and Drug Administration approved IRE for clinical use in 2009 (7, 8), many studies on IRE treatment of solid tumors in the liver, pancreas, prostate, kidney and other sites have been published. China also approved IRE for ablating liver and pancreas tumors in June 2015.

The IRE-based ablation therapeutic apparatus used in the present study incorporated electrode probes that transmitted high-voltage direct current pulses from the generator to the tissue to ablate the target lesion (9). Mechanically, as an IRE-based device, the IRE therapeutic device instantaneously generates a sudden IRE electric field between positive and negative electrodes that penetrates cancer cell membranes between and near the electrodes to produce nanoscale micro-perforations of cell membrane surfaces. The ultimate effect is disruption of the homeostasis of the cellular environment which rapidly leads to cell death (10–12). Based on the characteristics of reversible electrical penetration of cells, electroporation therapy has been proposed as a novel therapy as cancer cells exhibit irrecoverable rupture and cell death, termed IRE (13). The present study evaluated the safety and efficacy of a new National Medical Products Administration (NMPA)-approved IRE-based device for ablation of malignant tumors of the liver and compared its effects on a RFA control group of patients who received RFA.

## 2 Materials and methods

### 2.1 Patients

A total of 152 patients aged 34 to 70 years, who had malignant tumors of the liver, were enrolled from 5 hospitals. The clinical trial adhered to the Declaration of Helsinki and relevant clinical trial specifications and regulations in China, and approval was obtained from the Ethics Committees of Chinese PLA General Hospital (approval number: 2018-012), Tianjin Medical University General Hospital, Tianjin First Central Hospital, Qingdao University Hospital, and Beijing Youan Hospital. Every patient provided written informed consent before participating in the trial which was submitted to the Chinese Clinical Trial Registry (ChiCTR1800017516). The inclusion criteria were: patients aged 18 to 70 years who had received a clinical and imaging diagnosis of liver malignancy; diameter of liver tumor ≤ 4 cm; number of liver lesions was ≤ 3; ECOG scores were ≤ 2 points; and survival expectancy was > 6 months.

The exclusion criteria were: patients who had developed bacteremia, toxemia or other serious infectious diseases; severe coagulation dysfunction; suffered from severe heart, brain, lung or other diseases; had cardiac pacemakers or defibrillators, electronic equipment and metal parts implanted; or had a history of epilepsy. Further details on the inclusion and exclusion criteria are given in the supplementary documents.

### 2.2 Study design and randomization

It was a randomized, parallel, positive controlled clinical trial. Through screening and evaluation, patients were randomly divided into RFA and IRE groups. Stratified permuted block randomization was achieved by a randomization specialist who generated random code according to the proportion of the RFA and IRE group using SAS^®^ ver. 9.4 statistical software. Each trial patient was provided with a random envelope, marked either as an IRE or RFA group. The patients were then each assigned a unique random number.

Each hospital that hosted the trial opened the sealed instructions according to the order of patients, then recorded the date and time of opening, signed the initiator and assigned them to the treatment device. All the patients enrolled in the IRE or RFA group were treated either with an IRE therapy device or a RFA system (Covidien 11c, USA).

### 2.3 Objectives of clinical evaluation

#### 2.3.1 Primary objective

The success rate of tumor ablation was the primary efficacy indicator, which was defined as the tumor ablation success rate in the IRE group 1 week after the ablation procedure.

#### 2.3.2 Secondary objectives

1) the after-ablation recurrence rate of liver tumor foci at 1 and 3 months in the RFA and IRE groups; 2) tumor ablation time; 3) the incidence of ablation related complications such as bleeding, infection, pancreatitis, gastrointestinal fistula, pancreatic fistula, bile duct injury or arrhythmias. The safety objectives were the incidence of adverse events (AEs), evaluated by follow-up laboratory investigations and image analyses.

### 2.4 Evaluation methods for the objective parameters

The primary efficacy outcome was evaluated as the success rate of tumor ablation. In the follow-ups, tumor ablation site(s) were revisited by imaging examinations either with enhanced CT or MRI, 1 week after ablation. The tumor was considered to be completely ablated if the residual lesion could not be visualized radiologically. If the lesion treatment produced complete tumor ablation, the procedure was considered to be successful. In addition, tumor ablation time was defined as the period from the initiation of ablation until the surgeon believed that the tumor was completely ablated.

*Secondary efficacy outcomes*: post-ablation recurrence of liver tumor was evaluated 1 month and 3 months after ablation using enhanced CT or MRI imaging. If no radiologically visualized tumor was found in the liver, the outcome was defined as no recurrence. The non-recurrence rate was calculated by the formula: *number of patients without recurrence/total number of patients treated × 100%*.

The ablation-related complications included: bleeding; infection; pneumothorax; pancreatitis; gastrointestinal perforation; fistula formation; bile duct or/and pancreatic duct injury; arrhythmias; and other lesser conditions. The incidence of complications was equal to *number of patients with complications / total number of patients treated × 100%*.

The safety of the new device was further explored based on related AEs, which were mathematically evaluated by calculating and comparing the incidence of AEs and the incidence of serious AEs (SAEs) for both the IRE and RFA groups.

#### 2.4.1 Equipment and preoperative preparation

The range of settings for IRE-based ablation (Intelligent Health Medical Co., Ltd, Tianjin, China) were: number of ablation electrodes: 2~6; pulse setting range: 1-10 groups with 1-10 pulses in each group; pulse voltage range: 1,000 V – 3,000 V; pulse duration: 90 μs or 100 μs; the maximum energy of a single pulse: 15 J; the maximum pulse current: ≤ 50 A; The device also had an ECG synchronization function (discharge delay range 300-500 ms). Multiple security protection settings were enabled when using the device thus (1): *Output current limit*: When the current value between the electrode ablation needles detected by the main engine exceeded the operating parameter range, the device automatically triggered an alarm to indicate that the current was too high, and automatically stopped pulse transmission to avoid the output energy exceeding the maximum current setting (2). *Double triggered foot switch*: included a system which permitted double triggering of a foot switch, which prevented the occurrence of pulse delivery accidents during surgery. The foot switch system required the user first to press the “ready” pedal switch to start the system, and then press the “start” pedal switch in sequence. Then the energy was transmitted to the patient within 10 s. If the system did not output a pulse for more than 10 s, the surgery was considered to be invalid. 3) *Test voltage*: after placing the ablation needles and before surgery, the main machine delivered a group of low voltage pulses of 300 V to ensure that the system worked normally; the electrode ablation needles were spaced at appropriate locations (4). *Anti-arcing design*: the device had a built-in current limiting resistor. If a short circuit occurred, the current would be released through the current limiting resistor to prevent arc generation (5). If other accidents occur and the surgery needed to be interrupted in an emergency, a pulse stopping button on the pulse generation interface or a red stopping button on the front of the apparatus could be pressed.

Patients were first diagnosed as having a hepatic malignant tumor by CT or MRI enhanced imaging. According to the preoperative location, shape and size of the lesions determined by CT and/or MRI enhanced images, combined with surgical experience, the surgery team determined the number of intraoperative ablation probes required. Two to 6 appropriate electrodes were positioned after the development of a preliminary planning scheme for the percutaneous path of the ablation electrode needles.

The basic principles were as follows (1): Due to sequential pulse discharge between electrode pairs during IRE ablation, it was important to ensure that the optimal ablation distance between the probes was 1.5~2.2 cm, and that the electrodes were parallel to each other (2). The ablation area should cover all lesions and exceed the edge of the lesions by 0.5 cm, so as to completely cover the whole lesion with a minimum number of needle electrodes (3). For irregular lesions, needles were placed along the long axis of the lesions on the premise of avoiding other organs and blood vessels as much as possible. After the first ablation, the needles can be pulled back to continue the next ablation. Overlapping ablation areas should not be too large, and the number of ablations in the same area should not exceed 3 to avoid thermal damage (4); When the lesion was close to other important structures such as blood vessels, the gallbladder or intestine, the tips of the electrode needles should not make contact with these structures vertically to prevent mechanical damage caused by discharging the pulses; and (5) The percutaneous path of the electrode needles should avoid being close to vascular walls, to prevent thermal damage due to the high-temperature of the electrode needle surface.

After intraoperative electrode needle puncture was completed, the vertical distance between the ablation electrode needle pair was measured again using computer image reconstruction. Then, the intraoperative parameter settings were adjusted according to the actual measurement structure, and the formal ablation was performed after the test current was deemed to be within a reasonable range.

#### 2.4.2 Treatment procedures

IRE: General anesthesia was conducted during the surgery. Anesthesia was inducted with midazolam, propofol, fentanyl, and rocuronium. Tracheal intubation was assisted by video laryngoscopy, after which anesthesia was maintained using combined intravenous and inhalation drugs (sevoflurane, propofol, remifentanil). In order to alleviate muscle spasm during ablation discharge, rocuronium with an induction dosage of 0.6 mL/kg was administered intravenously during anesthesia at the beginning of the surgery. After the electrode probe was punctured in place, a top-up dose of 0.6 mL/kg rocuronium was administered again before ablation discharge was started. During surgery, a needle was punctured following the preoperative plan under the guidance of imaging. After the puncture was in place, a 3D reconstruction image was used to show the relative location of the electrode in the cross section of the ablation probe and the distance between the electrode pair was measured. Ablation parameters were selected and tested according to the effective ablation distance of the needle electrodes. The liver is rich in water content and relatively uniform in texture. The initial exposed tip length of an electrode was generally set at 2-2.5 cm. Because hilar liver vessels are abundant and have uneven structure, generally the exposed tip length was adjusted to 1.5-2 cm to avoid an uneven electric field that may affect the ablation effect. For the ablation parameters, the electric field intensity was usually set between 1,500 and 2,800 V/cm, which could be adjusted according to the tip distance with pulse numbers of 70 to 100 at a pulse width of 90 or 100 μs. After 10 to 20 pulses were tested, the trend of the current was determined. If the current was > 25 A and showed a gradual rising trend and was < 45 A, the parameters were reasonably selected and the pulse could be formally applied. Otherwise, the parameters were adjusted and tested again until the ablation was in a reasonable area before formal ablation was carried out. Formal ablation was performed with ECG monitoring, that involved a synchronous ECG monitoring device to identify the absolute refractory period of the cardiac cycle to ensure electrical pulses were released during the refractory period. However, in patients with a history of arrhythmia or with lesions located subdiaphragmatically, the potential increased risk of IRE causing arrhythmia should be noted and preparations made accordingly.

**Figure 1:**
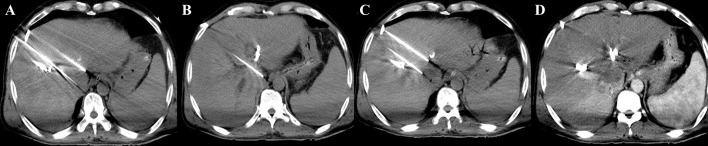
IRE procedure **(A, B)** During the operation, four 19G ablation probes were used to puncture the edges of the lesion. The active tip length (ablation area) was 15 mm, the voltage-to-distance ratio was 1,500-1,800 V/cm, and the pulse length was 90 μs. **(C)** The probes were pulled back twice to make segmental ablation. **(D)** Immediate postoperative enhanced scan showed decreased enhancement in the ablation area and a scattered gas density shadow, and no damage was found in the surrounding portal vein structure.

**Figure 2:**
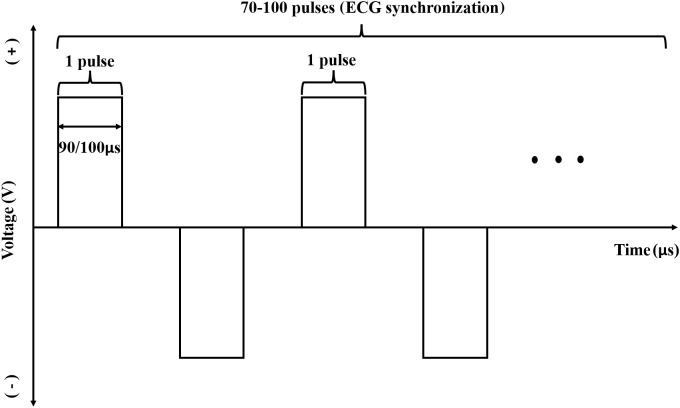
Schematic diagram of pulse waveform used with the device.A single pulse width was 90 μs or 100 μs and the number of ablation pulses in a single group was generally 70-100. The pulse was released during the absolute refractory period of the cardiac cycle with synchronous detection by ECG in the whole ablative period. In the ablation process, the pulses were discharged from positive and negative direction alternatively, and the absolute value of pulse discharge voltage in one group was the same.

Under CT-guidance, high-voltage direct current pulses were transmitted to a certain number (≥ 2) of electrode probes, which were percutaneously inserted at the edge of the lesion (**Figures 1A, B**) and ablation of target lesions conducted using IRE according to the preoperative treatment plan. This device was designed with a dual-electrode operation mode, with positive and negative probes, and a switch during ablation. Up to 6 electrodes could be connected and positioned in soft tissue at fixed distances to achieve the effect of several dual-electrode configurations, to achieve uniformity of ablation. Then, a CT scan was made to confirm the location of the needle electrodes. The vertical distance between the ablation electrode needle pair was measured after image reconstruction. The voltage, pulse numbers and times of needle pull back were set (**Figures 1C**) according to the size of the lesion and the distance between the needles. Patients were resuscitated at the end of the ablation procedure. An imaging enhancement examination was performed one week and one month after surgery (**Figures 1D**). The parameters during IRE interventions were: number of electrode ablation needles used, 2-6; number of ablation electrode pairs, 1-7; number of needle withdrawal times, 0-3; voltage, 2,000-3,000; number of pulse groups, 10-240; total number of pulses, 100-2,400; single pulse duration (μs), 90-100; active lengths of probes (cm), 1.5-3; currents (A), 17-48 (**Table S1**). **Figure 2** shows a schematic diagram of the pulse waveforms.

RFA: After local anesthesia using 1% lidocaine injection, a certain number of ablation needles were inserted percutaneously into the lesion according to the preoperative plan. Then a CT scan was performed to confirm the location of the needles and then the ablation time and power were set. The needles were withdrawn at the end of the ablation procedure. An imaging enhancement examination was carried out 1 week and 1 month after surgery.

*Differences between IRE and RFA*: The treatment area of the IRE device can be returned to normal function. IRE induces cell death within the targeted tissue through a series of electric pulses that elevate the transmembrane potentials to an extent that permanently damages the lipid bilayers throughout the treated region. In contrast, RFA denatures lesion protein by hyperthermia, which leads to coagulation, necrosis and the tissue structure no longer exists in the ablation area. The tissue remaining is necrotic and toxic to organs and cannot function normally again.

#### 2.5 Sample size

Based on previous clinical reports and present practices, the success rate of tumor ablation 1 week after surgery in the IRE group was expected to be 95%. After discussion, the non-inferiority margin was set at 10%, and the significance level of the hypothesis test was set as α = 0.025 for a single tail with a power of 80% (1-β). This test was applied to estimate the sample size required for each group (74 patients per group).

### 2.6 Statistical analysis

Efficacy analysis was performed on the per-protocol and full-analysis sets, baseline demographic data on the full-analysis set only, and safety evaluations using the safety set. Furthermore, the number of patients, mean, standard deviation, median, minimum and maximum were calculated for the description of quantitative indicators. A *t*-test or Wilcoxon signed rank test was used when appropriate. Categorical values were statistically analyzed with a chi-squared test or Fisher’s exact test. Wilcoxon rank-sum or CMH tests were employed to assess grading data. Statistical analyses were performed using a two-tailed test and a *P*-value ≤ 0.05 was considered to be statistically significant; 95% was taken as the CI. All statistical analyses were carried out using SAS software (ver. 9.4).

## 3 Results

### 3.1 Demographic baseline and clinical characteristics

**Figure 3:**
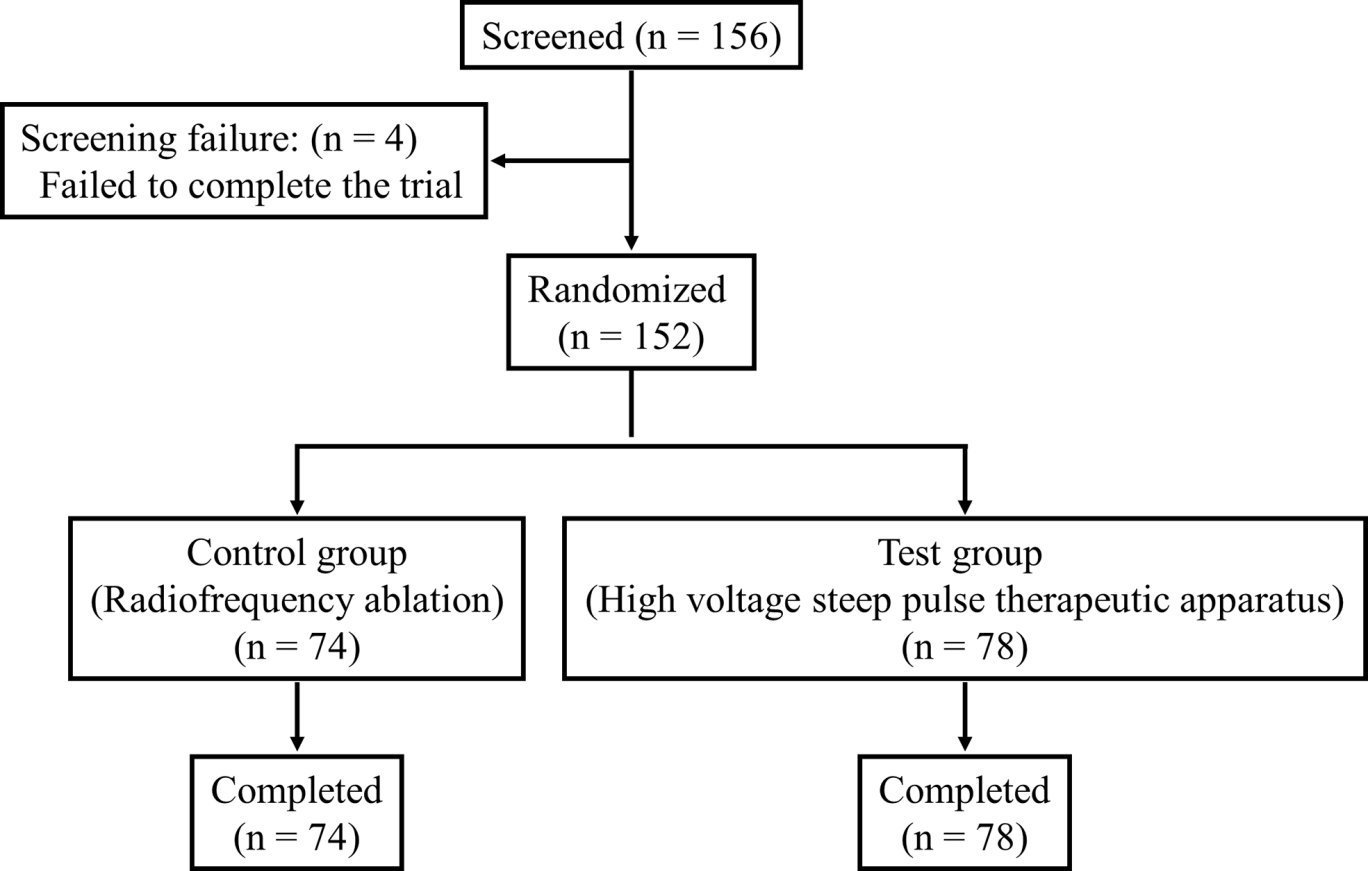
Flowchart of the study.

A total of 156 patients after randomization were enrolled in the trial, of which 152 completed the trial and 4 (2.63%) failed to do so. Following further stratification, 78 patients in the IRE group completed the trial, while 74 patients completed the trial in the RFA group (drop-out rate, 5.13%) (**Figure 3**).

The average age of patients was 58.88 ± 7.95 years in the IRE group and 57.29 ± 8.40 years in the RFA group. In the IRE group, 78 (100.00%) patients had a history of liver cancer, including 56 (71.79%) with hepatocellular carcinoma and 22 (28.21%) with liver metastases; the average maximum diameter of tumors was 2.31 ± 0.85 cm. Most patients [76 (97.44%)] had ECOG scores between 0 and 1. In the RFA group, 74 (100.00%) patients were enrolled, including 55 (74.32%) with primary liver cancer and 19 (25.68%) with liver metastases. The average maximum diameter of tumors was 2.11 ± 0.82 cm in this group, and the 74 patients (100%) all had ECOG scores between 0 and 1 (**Table 1**). There were no differences in the demographic baseline and tumor characteristics between the 2 groups.

**Table 1:** Demographic baseline and clinical characteristics of the patients.

Variable	IRE group	RFA group	Total	Statistic	*P*-value
**Age**					
N	78	74	152	Wilcoxon rank sum test	0.263
Mean ± SD	58.88 (7.95)	57.29 (8.40)	58.11 (8.19)		
**Gender, n (%)**					
Male	61 (78.21%)	59 (79.73%)	120 (78.95%)	Chi-squared test	0.818
Female	17 (21.79%)	15 (20.27%)	32 (21.05%)		
**BMI (kg/m^2^, mean ± SD)**	23.91 (2.98)	24.18 (3.87)	24.05 (3.44)	Wilcoxon rank sum test	0.691
**Tumor diagnostics**					
Liver cancer, n (%)	78 (100.00%)	74 (100.00%)	152 (100.00%)		
Primary liver cancer, n (%)	56 (71.79%)	55 (74.32%)	111 (73.03%)	Chi-squared test	0.725
Liver metastases, n (%)	22 (28.21%)	19 (25.68%)	41 (26.97%)	Chi-squared test	0.725
**Max diameter (cm, mean ± SD)**	2.31 (0.85)	2.11 (0.82)	2.21 (0.84)	Wilcoxon rank sum test	0.086
**Number of lesions**					
1, n (%)	54 (69.23%)	49 (66.22%)	103 (67.76%)	CMH test	0.466
2, n (%)	20 (25.64%)	18 (24.32%)	38 (25.00%)		
3, n (%)	4 (5.13%)	7 (9.46%)	11 (7.24%)		
**ECOG score**					
0, n (%)	68 (87.18%)	62 (83.78%)	130 (85.53%)	CMH test	0.898
1, n (%)	8 (10.26%)	12 (16.22%)	20 (13.16%)		
2, n (%)	2 (2.56%)	0 (0.00%)	2 (1.32%)		
Patients with comorbidities, n (%)^*^	78 (100.00%)	74 (100.00%)	152 (100.00%)	Chi-squared test	–

*includes various cardiac, hepatic, pulmonary, renal, gastrointestinal, endocrine and metabolic diseases.

### 3.2 Primary efficacy

A comparison showed that the success rate of liver tumor ablation of 94.87% in the IRE group was not significantly different from 95.95% in the RFA group (*P* = 0.761). The 95% CI of the difference in the success rate of the 2 different tumor ablation methods was between -7.72% and 5.57%. The lower limit of the CI was significantly greater than -10% with *P* = 0.006. Therefore, the new ablation device in the IRE group was considered non-inferior to the RFA group (**Table 2**).

**Table 2:** Success rate of liver malignant tumor ablation (%).

		IRE group	RFA group	*P*-value
**Ablation success rate**	n% (N)	74 (94.87%)	71 (95.95%)	0.761
	Difference of success rate	-1.07%		0.004
	95% CI	-7.72%, 5.57%		

A CMH test that corrected for the center effect was employed; Difference of success rate = IRE - RFA. The IRE for non-inferiority was one-tailed with a non-inferiority margin of -10%.

### 3.3 Secondary efficacy

The recurrence rates after ablation at 1 month and 3 months were 0.00% and 13.89% in the IRE group and 2.90% and 7.25% in the RFA group respectively but the recurrence rates in the two groups was not significantly different (*P* = 0.228, *P =* 0.201). In addition, the average ablation time in the IRE group was 34.29 ± 30.38 min, which was significantly different from the time of 19.91 ± 16.08 min in the RFA group (*P* < 0.001) (**Table 3**).

**Table 3:** Comparison of secondary efficacy between the two groups.

		IRE group	RFA group	*P*-value
**Recurrence rate**				
1-month post-op	Y, n (%)	0/77 (0.00%)	2/73 (2.90%)	0.228
3-months post-op	Y, n (%)	10/72 (13.89%)	5/69 (7.25%)	0.201
≥ 6 months post-op	Y, n (%)	10/75 (13.33%)	14/71 (19.72%)	0.320
**Tumor metastasis**				
≥ 6 months post-op	Y, n (%)	7/75 (9.33%)	6/71 (8.45%)	0.852
**Death**				
≥ 6 months post-op	Y, n (%)	9/76 (11.84%)	8/71 (11.27%)	0.856
**Ablation time (min)**	Pt Num	78	74	
	Mean (SD)	34.29 (30.38)	19.91 (16.08)	< 0.001
	Median	28.00	12.50	
	Min, max	2.00, 220.00	5.00, 93.00	
Complications after operation**	Pt Num	77	72	
1-week post-op	Y, n (%)	5 (6.49)	5 (6.94%)	1.000
1-month post-op	N, n (%)	1 (1.30%)	2 (2.78%)	0.610
3-months post-op	N, n (%)	0 (0.00%)	1 (1.43%)	0.490

**The complications included all intra-ablation AEs.

In the IRE group, 5 (6.49%) patients had complications 1 week after the operation; 1 (1.30%) patient had complications at 1 month and 0 (0.00%) patients had complications after 3 months. However, in the RFA group, 5 (6.94%) patients had complications 1 week after the operation, 2 (2.78%) at 1 month and 1 (1.43%) at 3 months. The differences between the two groups were not significantly different (all *P* > 0.05) (**Table 3**).

After 6 months follow-up, the incidence of recurrence or detection of metastasis in the liver cancer patients were 13.33% (10/75) and 9.33% in the IRE group and 19.72% and 8.45% in the RFA group (*P =* 0.320, *P =* 0.852), respectively. The survival rate after 6 months follow-up was 88.16% in the IRE group and 88.73% in the RFA group. For both groups, no significant differences were found in the secondary efficacy parameters.

### 3.4 Safety

The safety of the device was evaluated in the whole cohort. In the IRE group, 51 patients developed 191 AEs (incidence rate 65.38%), while 54 patients had 184 AEs in the RFA group (incidence rate 72.97%). SAEs were observed in 9 patients and 22 AEs in the IRE group (11.54%), while 16 patients and 31 AEs (21.62%) occurred in the RFA group. However, there were no significant difference in the incidences of AEs or SAEs between the 2 groups (**Table S2**). Seven patients experienced 8 treatment-related AEs (7.85%) in the IRE group, the most common being edema of the limbs and fever. Edema of the limbs was of a mild grade, while fever could be severe. Other events were subcapsular hemorrhage of the liver, elevated alanine aminotransferase, postoperative hemocholecyst, postoperative thrombosis, postoperative residual, although they were all of mild grade. However, it is noteworthy that the RFA group of patients did not exhibit any device-related AEs or SAEs (**Table 4**) (*P* < 0.01).

**Table 4:** Summary of treatment related adverse events.

Treatment-related AEs category, SOC/PT	IRE/RFA	Number of patients (N)	Number of AEs (n)	Grade, N (n)		
				Mild	Moderate	Severe
**Hepatobiliary disorders**						
Subcapsular hemorrhage of liver	1/0	1	1	1 (1)		
**General disorders and administration site conditions**						
Edema of limbs	1/0	1	2	1 (2)		
Fever	1/0	1	2			1 (2)
**Investigations**						
Elevated alanine aminotransferase	1/0	1	1		1 (1)	
**Injury, poisoning and procedural complications**						
Postoperative hemocholecyst	1/0	1	1	1 (1)		
Postoperative thrombosis	1/0	1	1	1 (1)		
Postoperative residual	1/0	1	1	1 (1)		

## 4 Discussion

As a new image-guided locoregional tumor ablation system, IRE possesses non-thermal characteristics to circumvent the heat-sink-effect and execute its therapeutic function in close proximity to critical anatomical structures such as the bile ducts or neurovascular bundles (14). The present study revealed a trend towards higher recurrence rates for tumors > 4 cm as well as for percutaneous access (15). Taken together, percutaneous access, tumor diameters > 2 cm and colorectal metastasis (CRM) are indicators associated with a higher risk of local failure of the procedure.

Overall, the use of IRE to treat hepatic malignancies was successfully initiated, with multifaceted clinical evidence mainly available for hepatocellular carcinoma and CRM (16–18). With regard to this evidence, IRE demonstrated favorable toxicity even when performed in proximity to sensitive anatomical structures, and the current literature suggests technical practicability and beneficial clinical outcomes. However, lesion size remains a limitation for the efficacy of IRE but can be countered by optimizing the number and configuration of the needles (19–21).

Previous clinical investigations reported that IRE therapy generally required only 100 ultra-short pulses of 90 µs or 100 µs between the 2 electrodes, when treating solid tumors ≤ 3 cm in diameter (22). Within only 2-3 min, the ablation procedure of an electrode pair can be completed. Moreover, this ablation not only delivers more precise and complete ablation to nodules in the liver, regardless of their location, size and shape, but also can avoid important anatomical structures such as blood vessels, nerves and biliary ducts in the ablation region. Experiments have shown that the ablation zone has a clear boundary and a demarcation thickness of only 1-2 cell units, which made the treated and untreated regions distinct, such that the effectiveness of treatment, outcomes and subsequent tracking can be more accurately evaluated (23). Traditional ablation denatures tissue by temperature damage followed by coagulative necrosis, which permanently damages tissue structure in the entire ablation region. In contrast, IRE therapy induces cell death by different mechanisms, namely necrosis and apoptosis, and also immunogenic cell deaths such as necroptosis and pyroptosis (24, 25). IRE therapy does not form scars or produce tissue coagulative necrosis. After surgery, other therapies can still be used to further enhance treatment if required, such as radiation therapy and chemotherapy.

In the present trial, we found using a novel IRE therapeutic device that compared to RFA, produced similar tumor ablation success, recurrence and post-ablation adverse effects rates in patients with liver cancer. Moreover, the liver nodules treated by IRE ablation had a comparable size to those treated with RFA. Thus, the IRE device achieved a comparable ablation effect on liver cancer to that of RFA. It should be noted that the ablation time of IRE was significantly prolonged, which may be related to the large number of discharge electrodes, unstable heart rates and interference of ECG synchronization during ablation.

However, to establish the safety profile for the new IRE ablation device, post-treatment AEs and complications are crucial parameters for further analysis. In comparison, the IRE device produced a better performance and a lower post-treatment AE rate than RFA (15.56% vs. 22.09%). Nevertheless, IRE produced several device-related complications, including fever, subcapsular hematoma of the liver, intra-gallbladder hemorrhage and severe edema, with rates of 6.67%, which were significantly different from RA. Among them, subcapsular hemorrhage and postoperative hemocholecyst may be caused by mechanical damage in the process of puncture adjustment in order to maintain the parallelism of the electrode needles. Other AEs, such as elevated ALT, thrombus related to great vein catheterization and edema of the lower extremities were included only because they could not be ruled out as unrelated to the device. One week after surgery, one patient had chest and back pain, and their body temperature was raised up to 38.6°C. Therefore, the investigators judged that it was an SAE and reported it again one month following surgery. After hospitalization, the patient’s condition improved and was discharged. An abdominal CT showed a flake-like fluid density shadow in the hilar region and around the head of the pancreas. This SAE was considered to be due to tumor necrosis and heat absorption after the treatment, but the possibility of infection could not be excluded.

There were several limitations to our study. First, an insufficient number of patients were enrolled. This is because most patients with malignant pathologies mentally hesitate to participate in any clinical trial to evaluate the safety and effectiveness of any novel treatment. Second, the patients in the study were not well stratified according to comorbidity and sites of lesion(s), which is crucial for evaluating selection criteria and potential injury to various intrahepatic structures. Third, the patients had not been followed-up for a longer period of time, meaning that the possible therapeutic benefit could not be fully evaluated.

In conclusion, this randomized study comparing two ablation methods and demonstrated that the novel IRE therapeutic device was not inferior to RFA, producing comparable tumor ablation success and recurrence rate, and AEs in patients with hepatic malignant tumors.

## Data availability statement

The raw data supporting the conclusions of this article will be made available by the authors, without undue reservation.

## Ethics statement 

The study involving human participants was reviewed and approved by the Ethics Committees of Chinese PLA General Hospital (approval number: 2018-012), Tianjin Medical University General Hospital, Tianjin First Central Hospital, Qingdao University Hospital, and Beijing Youan Hospital. The patients/participants provided their written informed consent to participate in this study.

## Author contributions

YX and XiaobZ was responsible for the conception and design of the study. All authors were responsible for acquisition and analysis of data; furthermore, XiaobZ and JinL were in charge of statistical analysis. XiaobZ drafted the manuscript; YX revised and commented on the draft. All authors contributed to the article and approved the submitted version.

## Conflict of interest

This clinical study was supported by funding from Intelligent Health Medical Co., Ltd, Tianjin, China. The funding body had no role in the design of the study or collection, analysis, interpretation of data, or writing of the manuscript. The authors declare that the research was conducted in the absence of any commercial or financial relationships that could be construed as a potential conflict of interest.

## Publisher’s note

All claims expressed in this article are solely those of the authors and do not necessarily represent those of their affiliated organizations, or those of the publisher, the editors and the reviewers. Any product that may be evaluated in this article, or claim that may be made by its manufacturer, is not guaranteed or endorsed by the publisher.

## Abbreviations

AEs, adverse events; CRM, colorectal metastasis; IRE, irreversible electroporation; NMPA, National Medical Products Administration; RFA, radiofrequency ablation; SAE, serious adverse event; TRAE, treatment related adverse event.
