# Supplementary material for: Novel irreversible electroporation ablation (Nano-knife) versus radiofrequency ablation for the treatment of solid liver tumors: a comparative, randomized, multicenter clinical study
Source: Front Oncol. 2022 Sep 29;12:945123. doi: 10.3389/fonc.2022.945123 (PMC9557230; doi:10.3389/fonc.2022.945123)
Supplement: Supplementary file 1 [file DataSheet_1.docx]

**Supplementary materials**

**Supplementary File 1. Inclusion and exclusion criteria**

*Inclusion criteria*

1) Male or female patients aged between 18 and 70 years

2) Patients had clinical and imaging diagnosis of liver malignancy

3) The maximum diameter of the liver tumor was ≤ 4 cm

4) The number of liver lesions was ≤ 3

5) ECOG scores of patients were ≤ 2 points

6) Expected survival of the patients was > 6 months

*Exclusion criteria*

Patients were not be allowed to participate in the trial if they met any of following criteria:

1) They developed bacteremia, toxemia or other serious infectious diseases

2) Had severe coagulation dysfunction

3) Suffered from severe heart, brain, lung or other diseases

4) Had cardiac pacemakers or defibrillators, electronic equipment and metal parts implanted

5) Had a history of epilepsy

6) Presented with arrhythmias

7) Diagnosed with acute myocardial infarction within 6 months

8) Patients who were pregnant or lactating, or those who planned to become pregnant within 1 year

9) Patients who had participated in any drug and/or medical device clinical trial within 3 months before enrollment in the present trial

10) Researchers who believed that other factors disqualified patients from or would affect their participation in the trial

**Table S1. Treatment parameters of the IRE patients (the data of 2 patients were incomplete and not listed in the table)**

| **Patient No.** | **Number of electrode ablation needles used** | **The number of ablation electrode pairs** | **Number of needle withdrawn times** | **Voltage-to-distance ratios (V/cm)** | **Number of pulse groups** | **Total number of pulses** | **Single pulse duration**  **(μs)** | **The active length of probe (cm)** | **Current (A)** |
| --- | --- | --- | --- | --- | --- | --- | --- | --- | --- |
| 1 | 5 | 6 | 0 | 1500.0-1866.7 | 50 | 500 | 90 | 2 | 35-40 |
| 2 | 2 | 1 | 1 | 1666.7-2000.0 | 15 | 150 | 90 | 2.5 | 28-39 |
| 3 | 4 | 2 | 1 | 1500.0-1666.7 | 40 | 400 | 90 | 2.5 | 29-40 |
| 4 | 2 | 1 | 0 | 2000.0 | 10 | 100 | 90 | 2.5 | 30-38 |
| 5 | 3 | 3 | 0 | 1500.0 | 30 | 300 | 90 | 2 | 35-46 |
| 6 | 2 | 1 | 0 | 1666.7 | 10 | 100 | 90 | 2 | 34-43 |
| 7 | 2 | 1 | 1 | 1500.0-2000.0 | 20 | 200 | 90 | 2, 2.5 | 30-40 |
| 8 | 2 | 1 | 1 | 1500.0 | 20 | 200 | 90 | 2.5 | 35-47 |
| 9 | 4 | 2 | 1 | 2000.0 | 40 | 400 | 90 | 2 | 29-42 |
| 10 | 3 | 3 | 1 | 1500.0 | 45 | 450 | 90 | 2 | 25-40 |
| 11 | 2 | 1 | 0 | 2000.0 | 10 | 100 | 90 | 2 | 17-20 |
| 12 | 2 | 1 | 1 | 2200.0 | 15 | 150 | 90 | 1.5 | 23-32 |
| 13 | 5 | 5 | 2 | 1500.0 | 100 | 1000 | 90 | 1.5, 2 | 33-44 |
| 14 | 3 | 3 | 1 | 1500.0 | 45 | 450 | 90 | 2, 2.5 | 25-32 |
| 15 | 2 | 1 | 2 | 1500.0 | 30 | 300 | 90 | 2.5 | 33-43 |
| 16 | 2 | 1 | 1 | 1875.0 | 20 | 200 | 90 | 2 | 29-34 |
| 17 | 3 | 3 | 1 | 1500.0-1866.7 | 45 | 450 | 100 | 2 | 34-42 |
| 18 | 3 | 3 | 3 | 1500.0-2000.0 | 150 | 1500 | 100 | 2 | 28-47 |
| 19 | 5 | 7 | 1 | 1500.0-1666.7 | 175 | 1750 | 100 | 2 | 30-47 |
| 20 | 2 | 1 | 0 | 1666.7 | 10 | 100 | 90 | 2 | 26-32 |
| 21 | 2 | 1 | 1 | 2500.0 | 15 | 150 | 90 | 2 | 25-38 |
| 22 | 2 | 1 | 0 | 2500.0 | 10 | 100 | 90 | 2 | 29-48 |
| 23 | 2 | 1 | 1 | 2500.0 | 20 | 200 | 90 | 1.5 | 26-35 |
| 24 | 3 | 3 | 1 | 1666.7 | 45 | 450 | 100 | 2 | 30-39 |
| 25 | 3 | 3 | 1 | 1500.0 | 45 | 450 | 90 | 2 | 31-44 |
| 26 | 5 | 4 | 1 | 1500.0-2307.7 | 60 | 600 | 100 | 2 | 30-48 |
| 27 | 2 | 1 | 1 | 2000.0 | 30 | 300 | 100 | 2 | 25-48 |
| 28 | 2 | 1 | 0 | 2000.0 | 10 | 100 | 90 | 1.5 | 28-42 |
| 29 | 2 | 1 | 0 | 1500.0 | 10 | 100 | 90 | 2 | 26-34 |
| 30 | 4 | 2 | 0 | 1500.0 | 20 | 200 | 90 | 2 | 30-45 |
| 31 | 2 | 1 | 1 | 1500.0 | 20 | 200 | 90 | 2 | 31-36 |
| 32 | 4 | 2 | 0 | 1500.0 | 20 | 200 | 90 | 1.5, 2 | 24-28 |
| 33 | 2 | 1 | 1 | 1666.7 | 20 | 200 | 100 | 2 | 24-43 |
| 34 | 2 | 1 | 2 | 1500.0 | 30 | 300 | 100 | 2 | 26-42 |
| 35 | 2 | 1 | 2 | 1500.0 | 30 | 300 | 90 | 2 | 22-28 |
| 36 | 2 | 1 | 1 | 2000.0 | 30 | 300 | 100 | 2 | 29-42 |
| 37 | 2 | 1 | 0 | 2000.0 | 10 | 100 | 90 | 1.5 | 25-40 |
| 38 | 2 | 1 | 1 | 1666.7 | 30 | 300 | 90,100 | 2 | 24-33 |
| 39 | 4 | 2 | 1 | 2000.0-2727.3 | 81 | 810 | 90 | 2 | 28-40 |
| 40 | 2 | 1 | 1 | 1666.7-1866.7 | 30 | 300 | 100 | 2 | 26-36 |
| 41 | 2 | 1 | 1 | 2000.0-2500.0 | 30 | 300 | 90 | 1.5 | 24-31 |
| 42 | 4 | 4 | 1 | 1500.0 | 80 | 800 | 100 | 1.5 | 20-36 |
| 43 | 2 | 1 | 1 | 1500.0 | 30 | 300 | 100 | 2 | 30-35 |
| 44 | 3 | 3 | 1 | 1500.0-1692.3 | 90 | 900 | 100 | 2 | 32-42 |
| 45 | 4 | 2 | 1 | 2000.0 | 60 | 600 | 90 | 2 | 17-32 |
| 46 | 2 | 1 | 0 | 1529.4 | 10 | 100 | 90 | 2 | 35-45 |
| 47 | 4 | 5 | 1 | 1529.4-1764.7 | 100 | 1000 | 90 | 2, 2.5 | 25-45 |
| 48 | 2 | 1 | 0 | 1500.0 | 10 | 100 | 90 | 3 | 39-46 |
| 49 | 4 | 4 | 0 | 1533.3-1666.7 | 40 | 400 | 90 | 2 | 33-44 |
| 50 | 2 | 1 | 1 | 1916.7 | 30 | 300 | 90 | 2 | 32-38 |
| 51 | 4 | 4 | 1 | 1500.0-2500.0 | 80 | 800 | 90 | 3 | 34-43 |
| 52 | 2 | 1 | 0 | 1733.3 | 10 | 100 | 90 | 2.5 | 32-39 |
| 53 | 2 | 1 | 0 | 1916.7 | 10 | 100 | 90 | 2.5 | 33-45 |
| 54 | 4 | 2 | 1 | 1866.7-2000.0 | 60 | 600 | 100 | 2 | 25-38 |
| 55 | 4 | 3 | 1 | 1866.7 | 60 | 600 | 90 | 2 | 28-45 |
| 56 | 6 | 3 | 1 | 1500.0-1666.7 | 60 | 600 | 90 | 2, 2.5 | 25-43 |
| 57 | 5 | 4 | 1 | 1555.6 | 60 | 600 | 90 | 2 | 30-42 |
| 58 | 5 | 4 | 1 | 1666.7-1764.7 | 60 | 600 | 90 | 2.5 | 32-40 |
| 59 | 4 | 4 | 1 | 1500.0-2000.0 | 120 | 1200 | 90 | 3 | 30-42 |
| 60 | 4 | 3 | 1 | 1500.0-1666.7 | 90 | 900 | 100 | 3 | 33-40 |
| 61 | 5 | 7 | 0 | 1578.9-1875.0 | 70 | 700 | 90 | 1.5 | 25-43 |
| 62 | 4 | 6 | 1 | 1500.0-1800.0 | 120 | 1200 | 90 | 1.5 | 26-47 |
| 63 | 4 | 6 | 2 | 1500.0-2000.0 | 90 | 900 | 90 | 1.5 | 19-45 |
| 64 | 4 | 5 | 1 | 1500.0-1833.3 | 100 | 1000 | 90 | 1.5, 2 | 23-38 |
| 65 | 4 | 4 | 2 | 1500.0-1764.7 | 120 | 1200 | 90 | 1.5 | 23-42 |
| 66 | 3 | 3 | 1 | 1500.0-1923.1 | 60 | 600 | 90 | 2 | 29-48 |
| 67 | 3 | 3 | 0 | 1923.1 | 30 | 300 | 90 | 2 | 24-45 |
| 68 | 4 | 5 | 1 | 1571.4-1625.0 | 100 | 1000 | 90 | 2 | 29-45 |
| 69 | 4 | 6 | 3 | 1666.7-2000.0 | 240 | 2400 | 90 | 1.5 | 19-40 |
| 70 | 5 | 6 | 1 | 1785.7-2153.8 | 120 | 1200 | 90 | 2 | 27-45 |
| 71 | 4 | 6 | 1 | 1647.1-2083.3 | 120 | 1200 | 90 | 2 | 26-36 |
| 72 | 4 | 5 | 1 | 1866.7-2083.3 | 100 | 1000 | 90 | 1.5 | 28-43 |
| 73 | 5 | 5 | 2 | 1500.0-2083.3 | 150 | 1500 | 90 | 2 | 30-44 |
| 74 | 2 | 1 | 0 | 2000.0 | 10 | 100 | 90 | 2 | 30-36 |
| 75 | 6 | 5 | 0 | 1666.7-1764.7 | 50 | 500 | 90 | 2 | 28-40 |
| 76 | 4 | 2 | 0 | 1875.0-2000.0 | 20 | 200 | 90 | 2 | 25-39 |

**Note:** Two patients who have incomplete data were not included in this table.

**Table S2. Summary of AEs that occurred**

| **SOC, PT** | **Number of patients (N)** | **Number of AEs (n)** | **Grade, N (n)** | | |
| --- | --- | --- | --- | --- | --- |
|  |  |  | **Mild** | **Moderate** | **Severe** |
| **Blood and lymphatic disorders** | | | | | |
| Anemia | 4 | 7 | 3 (6) | 1 (1) |  |
| Arrest of bone marrow | 2 | 3 | 1 (1) | 1 (2) |  |
| Thrombocytopenia | 2 | 2 | 2 (2) |  |  |
| **Cardiac disorders** | | | | | |
| Aortic stenosis | 1 | 1 |  | 1 (1) |  |
| Atrial fibrillation | 1 | 1 | 1 (1) |  |  |
| Coronary artery disease | 1 | 1 |  | 1 (1) |  |
| **General disorders and administration site conditions** | | | | | |
| Ache | 2 | 2 | 1 (1) |  | 1 (1) |
| Ascites | 2 | 3 |  | 2 (3) |  |
| Edema of limbs | 1 | 2 |  | 1 (2) |  |
| Fatigue | 5 | 8 | 5 (8) |  |  |
| Fever | 14 | 20 | 8 (11) | 1 (1) | 5 (8) |
| **Hepatobiliary disorders** | | | | | |
| Acute cholangitis | 4 | 4 | 2 (2) | 1 (1) | 1 (1) |
| Cholangitis | 2 | 2 | 2 (1) |  |  |
| Gallstones | 2 | 4 | 1 (1) | 2 (3) |  |
| Hepatalgia | 6 | 6 | 6 (6) |  |  |
| Hepatic insufficiency | 5 | 9 | 4 (7) | 1 (2) |  |
| Hepatorrhagia | 2 | 5 | 1 (3) | 1 (2) |  |
| Liver cirrhosis | 2 | 3 | 1 (1) |  | 1 (2) |
| Obstructive jaundice | 1 | 1 |  |  | 1 (1) |
| Right upper quadrant discomfort | 8 | 9 | 8 (9) |  |  |
| Subcapsular hemorrhage of liver | 1 | 1 | 1 (1) |  |  |
| **Immune system disorders** | | | | | |
| Allergy | 2 | 3 |  | 1 (2) | 1 (1) |
| Lupus erythematosus | 1 | 2 |  | 1 (2) |  |
| **Infections and infestations** | | | | | |
| Abdominal infection | 1 | 3 |  |  | 1 (3) |
| Acute exacerbations of chronic bronchitis | 1 | 1 | 1(1) |  |  |
| Biliary tract infection | 1 | 1 |  | 1 (1) |  |
| Chronic pharyngitis | 1 | 1 | 1(1) |  |  |
| Hordeolum | 1 | 1 | 1(1) |  |  |
| Infective fever | 1 | 3 |  |  | 1 (3) |
| Infection of abdominal wall incisional | 1 | 2 |  | 1 (2) |  |
| Pulmonary infection | 1 | 1 | 1(1) |  |  |
| Upper respiratory infection | 2 | 2 | 2(2) |  |  |
| Urinary tract infection | 1 | 1 |  |  | 1 (1) |
| **Injury, poisoning and procedural complications** | | | | | |
| Abdominal wound disruption | 1 | 2 |  |  | 1 (2) |
| Intraoperative  hemorrhage | 4 | 4 |  | 4 (4) |  |
| Intraoperative  hypertension | 1 | 1 |  | 1 (1) |  |
| Postoperative  hemocholecyst | 1 | 1 | 1 (1) |  |  |
| Postoperative residual | 3 | 3 | 2 (2) |  | 1 (1) |
| Nerve injury | 1 | 2 | 1 (2) |  |  |
| Postoperative thrombosis | 4 | 4 | 4 (4) |  |  |
| **Investigations** | | | | | |
| Abnormal blood  coagulation | 1 | 1 | 1 (1) |  |  |
| Decreased blood platelet | 2 | 2 | 1 (1) | 1 (1) |  |
| Elevated alanine  aminotransferase | 11 | 11 | 10 (10) | 1 (1) |  |
| Elevated blood pressure | 1 | 1 | 1 (1) |  |  |
| Elevated total bilirubin | 1 | 2 | 1 (2) |  |  |
| Hyperbilirubinemia | 1 | 1 | 1 (1) |  |  |
| Urinary protein | 1 | 1 |  | 1 (1) |  |
| **Metabolism and nutrition disorders** | | | | | |
| Loss of appetite | 14 | 15 | 14 (15) |  |  |
| Hyperchloremia | 1 | 1 | 1 (1) |  |  |
| Hyperammonemia | 2 | 2 | 2 (2) |  |  |
| Hypernatremia | 1 | 1 | 1 (1) |  |  |
| Hypoalbuminemia | 10 | 17 | 10 (17) |  |  |
| Hypochloridemia | 5 | 8 | 5 (8) |  |  |
| Hypokalemia | 5 | 5 | 5 (5) |  |  |
| Hyponatremia | 4 | 6 | 4 (6) |  |  |
| **Musculoskeletal and connective tissue disorders** | | | | | |
| Backache | 1 | 1 | 1 (1) |  |  |
| **Neoplasms benign, malignant and unspecified (including cysts and polyps)** | | | | | |
| Primary tumor | 5 | 8 |  | 2 (4) | 3 (4) |
| Recurrent neoplasm | 13 | 15 | 4 (4) | 7 (8) | 2 (3) |
| **Renal and urinary disorders** | | | | | |
| Dysuria | 1 | 1 | 1 (1) |  |  |
| Urinary tract discomfort | 1 | 1 | 1 (1) |  |  |
| Urodynia | 1 | 1 | 1 (1) |  |  |
| **Gastrointestinal disorders** | | | | | |
| Abdominal distension | 21 | 25 | 19 (23) | 2 (2) |  |
| Abdominal pain | 31 | 42 | 22 (29) | 9 (13) |  |
| Acid reflux/heartburn | 3 | 4 | 2 (3) | 1 (1) |  |
| Constipation | 2 | 3 | 2 (3) |  |  |
| Diarrhea | 2 | 3 | 1 (2) | 1 (1) |  |
| Dyspepsia | 1 | 2 | 1 (2) |  |  |
| Gastrointestinal  hemorrhage | 2 | 3 |  | 1 (2) | 1 (1) |
| Intestinal obstruction | 1 | 1 |  |  | 1 (1) |
| Abdominal discomfort | 1 | 1 | 1 (1) |  |  |
| Pancreatitis | 1 | 2 |  | 1 (2) |  |
| Pyloric obstruction | 1 | 1 |  |  | 1 (1) |
| Nausea | 13 | 13 | 13 (13) |  |  |
| Vomiting | 11 | 11 | 11 (11) |  |  |
| **Respiratory, thoracic, and mediastinal disorders** | | | | | |
| Chest tightness | 7 | 9 | 7 (9) |  |  |
| Chest pain | 1 | 1 | 1 (1) |  |  |
| Cough | 3 | 5 | 3 (5) |  |  |
| Hoarseness | 1 | 1 | 1 (1) |  |  |
| Left pleural thickening | 1 | 1 | 1 (1) |  |  |
| Pneumothorax | 5 | 9 | 5 (9) |  |  |
| Pneumonia | 1 | 1 | 1 (1) |  |  |
| Respiratory acidosis | 1 | 1 | 1 (1) |  |  |
| **Skin and subcutaneous tissue disorders** | | | | | |
| Skin rash | 1 | 3 |  | 1 (3) |  |
| **Vascular disorders** | | | | | |
| Hypertension | 2 | 2 | 1 (1) | 1 (1) |  |
